# Seizures as the only presentation of Pulmonary Embolism in the absence of Respiratory Symptoms

**DOI:** 10.12669/pjms.40.7.9426

**Published:** 2024-08

**Authors:** Omair Farooq, Amna Maqsood, Muhammad Ahmad, Muhammad Ibrahim Sohail, Maryam Rashid

**Affiliations:** 1 Omair Farooq Assistant Professor, Medicine Department, Farooq Hospital Lahore; 2 Amna Maqsood Demonstrator Pharmacology, Akhtar Saeed Medical and Dental College, Lahore - Pakistan; 3 Muhammad Ahmad Demonstrator Pharmacology, Akhtar Saeed Medical and Dental College, Lahore - Pakistan; 4 Muhammad Ibrahim Sohail Demonstrator Pharmacology, Akhtar Saeed Medical and Dental College, Lahore - Pakistan; 5 Maryam Rashid Professor Pharmacology, Akhtar Saeed Medical and Dental College, Lahore - Pakistan

**Keywords:** Seizures, Pulmonary Embolism, CT Angiogram

## Abstract

Pulmonary embolism is a life-threatening emergency. Seizure as the clinical presentation of pulmonary embolism is extremely rare. In this case report a 47-year-old female had an episode of seizure after undergoing total abdominal hysterectomy with bilateral salpingo-oophorectomy due to myometrial uterine fibroids. The patient had no past history of seizure or cardiovascular disease. Based on raised D-Dimers and echocardiography a provisional diagnosis of pulmonary embolism was made, which was confirmed on CT angiogram that showed bilateral saddle pulmonary embolism. Clinicians need to be aware that Pulmonary embolism is a possibility as the differential diagnosis for unexplained, new-onset of seizure activity.

## INTRODUCTION

Pulmonary Embolism is a potentially fatal disease that results in significant morbidity and mortality. A pulmonary embolism occurs when a thrombus breaks free, most often a blood clot, passes through the heart chambers into the pulmonary vasculature, and lodges in the pulmonary arteries.^1^ Clinical presentation of pulmonary embolism varies diversely from being asymptomatic, with nonspecific symptoms such as mild chest pain, slight dyspnea, and anxiety, or can be presented in its severe form with sudden cardiac arrest. Anticoagulation therapy with direct oral anticoagulants improves the outcome.^2^

Central nervous system (CNS) complications of pulmonary embolism include acute ischemic cerebrovascular events, venous thromboembolism in brain tumours and intracranial haemorrhage, while peripheral nervous system (PNS) complications of pulmonary embolism include neuropathic pain and peripheral neuropathy. Atypical neurological presentations of pulmonary embolism include seizures and syncope.^3^

After treatment, pulmonary embolism is still the cause of almost 1.3% of deaths in the United States, and without treatment, this number goes up to 30%. New onset seizures are an extremely unusual presentation secondary to acute pulmonary embolism and without proper identification and management, this condition can be fatal.^4^

This case report aims to highlight a rare presentation of new-onset seizures as a consequence of pulmonary embolism in a post-operative patient after a total abdominal hysterectomy with bilateral salpingo-oophorectomy. Initially, the challenging part was the lack of typical symptoms of pulmonary embolism.

## CASE REPORT

A 47-year-old female patient presented at Farooq Hospital (West Wood branch) with complaints of heavy menstrual bleeding with clots and dysmenorrhea associated with urinary urgency, nocturia, and dyspepsia for two years. On examination, the abdomen was soft and a large mass with irregular margins reaching up to epigastrium (32 weeks size) was palpable. Ultrasound of the abdomen and pelvis revealed a bulky uterus with uterine fibroids within the myometrium. Based on ultrasound, a plan of total abdominal hysterectomy with bilateral salpingo-oophorectomy was made. Her preoperative blood tests were insignificant including coagulation profile, thyroid profile and Troponin test. MRI pelvis without contrast showed a huge sub-serosal uterine fibroid measuring 19.5x12.4x15 cm with cystic degeneration. A total abdominal hysterectomy and bilateral salpingo-oophorectomy with ureteric stenting were carried out.

On the 3rd postoperative day, she suffered a single episode of generalized tonic-clonic seizures, and the patient was transferred to ICU. Her blood tests including CBC, D-Dimers and Troponin were sent which revealed severe anemia and raised D-Dimers up to 5.17 μ/ml. A provisional diagnosis of Pulmonary embolism was made but she showed no typical symptoms like chest pain, breathlessness and cough. Her vitals showed a heart rate of 130 bpm, respiratory rate of 26 breaths per minute and oxygen saturation was 98%.

**Fig.1:**
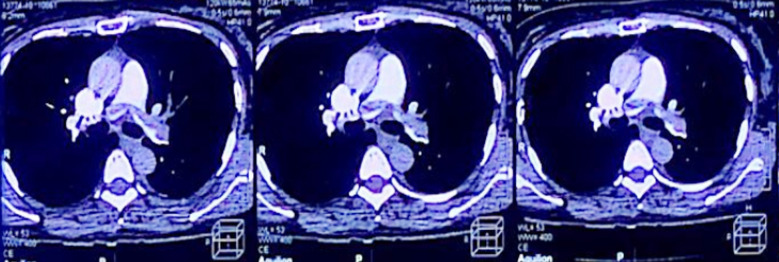
CT Angiogram, Showing bilateral saddle pulmonary embolism.

Serum electrolytes were unremarkable. ECG showed diffuse T wave changes and her troponin test was positive. Her Echocardiography revealed dilatation of the right atrium and right ventricle. CT pulmonary angiogram with contrast was carried out which showed bilateral extensive saddle pulmonary embolism as shown in (Fig.1). Her blood tests were repeated which showed a further increase in D-Dimers. Therefore, anticoagulation with heparin 1000 units per hour was commenced. Initially, a bolus of 80 units per kg/hr was given. Embolectomy and thrombolysis were contraindicated because of the risk of bleeding. On 4th postoperative day, patient was shifted to direct oral anticoagulant (rivaroxaban 15 mg BD). Her respiratory rate improved gradually, and tachycardia settled. Serial echocardiography done one day before discharge showed improvement in the right heart strain. She remained admitted for three more days with a total of seven days post operatively.

On follow-up one week after discharge, she was mobile and doing well and she had no further episode of seizure. Anti-Epileptics were not started as the likely cause of seizure in this case was pulmonary embolism leading to reduced cardiac output, reduced blood flow through the main pulmonary trunk and reduced cerebral perfusion leading to generalized tonic-clonic seizures. Rivaroxaban was reduced to 20 mg Once daily after 21 days for life.

## DISCUSSION

In retrospect, this case very much represented a rare presentation for pulmonary embolism (PE) as a sudden generalized tonic–clonic seizure in the setting of no previous risk factors. Seizures are well-known clinical neurological manifestations of different pathologies. They have been observed in cardiopulmonary diseases such as long QT syndrome, severe bradycardia, systemic hypertension, aortic dissection, and cardiac arrest.^5^-^7^ The pathophysiology behind seizure as the presenting sign for massive PE is well described by Marine and Goldhaber^8^, who reported two cases and suggested that massive PE caused transient right ventricular failure, and decreased cardiac output, causing transient global cerebral hypo-perfusion. PE can be caused by different etiologies, including thromboembolism, air embolism, fat embolism, septic embolism, amniotic fluid embolism, foreign material pulmonary embolism, and tumor embolism. In our reported case, DVT as well as PE was found. Thus, pulmonary arterial circulation was embolized by a DVT clot, which led to myocardial hypoxic injury and ischemic hypoxic encephalopathy.

Finally, the patient presented with a seizure. It is important to note, embolectomy and thrombolysis was contraindicated in our patient due to the recent major abdominal surgery and risk of bleeding. Anticoagulation, first with heparin and later with rivaroxaban was done. In summary, the clinician needs to be aware of all cardiovascular possibilities as explanations for sudden syncope with new onset of generalized seizure activity as shown in this case.

### Authors Contribution:

**OF, MA, AM:** Data Collection and Manuscript writing.

**MIS, MR:** Edited, Reviewed and did final approval of manuscript.

**OF:** Conceived and Drafted the Manuscript.

**MA:** Is responsible for the accuracy of the study.
